# COVID-19Base v3: Update of the knowledgebase for drugs and biomedical entities linked to COVID-19

**DOI:** 10.3389/fpubh.2023.1125917

**Published:** 2023-03-06

**Authors:** Syed Abdullah Basit, Rizwan Qureshi, Saleh Musleh, Reto Guler, M. Sohel Rahman, Kabir H. Biswas, Tanvir Alam

**Affiliations:** ^1^College of Science and Engineering, Hamad Bin Khalifa University, Doha, Qatar; ^2^International Centre for Genetic Engineering and Biotechnology (ICGEB), Cape Town Component, University of Cape Town, Cape Town, South Africa; ^3^Department of Pathology, Division of Immunology and South African Medical Research Council (SAMRC) Immunology of Infectious Diseases, Institute of Infectious Diseases and Molecular Medicine (IDM), Faculty of Health Sciences, University of Cape Town, Cape Town, South Africa; ^4^Wellcome Centre for Infectious Diseases Research in Africa, Institute of Infectious Diseases and Molecular Medicine (IDM), Faculty of Health Sciences, University of Cape Town, Cape Town, South Africa; ^5^Department of Computer Science and Engineering, Bangladesh University of Engineering and Technology, Dhaka, Bangladesh; ^6^College of Health and Life Sciences, Hamad Bin Khalifa University, Doha, Qatar

**Keywords:** SARS-CoV-2, CORD-19, deep learning, machine learning, COVID-19

## Abstract

COVID-19 has taken a huge toll on our lives over the last 3 years. Global initiatives put forward by all stakeholders are still in place to combat this pandemic and help us learn lessons for future ones. While the vaccine rollout was not able to curb the spread of the disease for all strains, the research community is still trying to develop effective therapeutics for COVID-19. Although Paxlovid and remdesivir have been approved by the FDA against COVID-19, they are not free of side effects. Therefore, the search for a therapeutic solution with high efficacy continues in the research community. To support this effort, in this latest version (v3) of COVID-19Base, we have summarized the biomedical entities linked to COVID-19 that have been highlighted in the scientific literature after the vaccine rollout. Eight different topic-specific dictionaries, i.e., gene, miRNA, lncRNA, PDB entries, disease, alternative medicines registered under clinical trials, drugs, and the side effects of drugs, were used to build this knowledgebase. We have introduced a BLSTM-based deep-learning model to predict the drug-disease associations that outperforms the existing model for the same purpose proposed in the earlier version of COVID-19Base. For the very first time, we have incorporated disease-gene, disease-miRNA, disease-lncRNA, and drug-PDB associations covering the largest number of biomedical entities related to COVID-19. We have provided examples of and insights into different biomedical entities covered in COVID-19Base to support the research community by incorporating all of these entities under a single platform to provide evidence-based support from the literature. COVID-19Base v3 can be accessed from: https://covidbase-v3.vercel.app/. The GitHub repository for the source code and data dictionaries is available to the community from: https://github.com/91Abdullah/covidbasev3.0.

## 1. Introduction

The global COVID-19 pandemic led to millions of deaths and imposed a huge burden on the economy worldwide (1). The vaccine rollout was not able to curb the fast spread of all COVID-19 variants (2), and vaccine availability is still far from what is required (3). As a result, there are still a considerable number of people infected with this novel coronavirus SARS-CoV-2 worldwide, which suggests that equal access to vaccines across the world needs to be reinforced, particularly in low- to middle-income countries (4). Therefore, there is an urgent need to repurpose well-tolerated existing drugs with high efficacy that can be easily administered to curb the disease progression of COVID-19. Hundreds of drugs have already been tested on COVID-19 patients, mostly in hospitals, resulting in a large amount of data of varying quality (5). The guidance issued globally followed this data to some extent; for example, the FDA recommended dexamethasone for hospitalized patients who require oxygen or mechanical ventilation, and the use of tocilizumab further improves survival. Recently, the FDA has approved the use of Paxlovid^TM^ (nirmatrelvir and ritonavir) as a prescribed drug for COVID-19; however, it is not free from side effects and concomitant use of Paxlovid^TM^ with statins may result in an adverse drug interaction (6). Therefore, the scientific community is still looking for effective therapeutic treatment options for COVID-19.

There are only a few studies available regarding the automated searching of COVID-19 drugs. The Chinese Antibody Society launched the “COVID-19 Antibody Therapeutics Tracker” (also called “Tracker”) program in March 2020 to track antibody-based COVID-19 intervention plans in preclinical and clinical development (7). This tracker presents the analysis and visualization of COVID-19 antibody development for over 150 research programs as of the 8th August 2020. The collected data is categorized by different targets and development status, as well as country of origin. Various public domain resources, such as published literature, preprints, official websites, news feeds, social media, and government databases, were also used for data collection.

A user-friendly platform (CO-19 PDB; COVID-19 Pandemic Data Base) for COVID-19 research was developed by Ullah et al. (8). A total of 59 databases were gathered into the CO-19 PDB database between December 2019 and May 2021 and the data was organized into six different categories, namely databases for (a) digital images, (b) genomic information, (c) literature collection, (d) visualization tools, (e) chemical structure, and (f) social science-related information. These databases concentrate on extracting a variety of information, such as genomic sequences, images, the latest news updates, reports, articles, and books. Yang et al. developed the “COVID-19 Antibody Therapeutics Tracker” to track antibody-based preclinical and clinical interventions globally to combat COVID-19 (7). The authors mentioned that two antibodies, CD6 and IL-6R, have been approved for the drugs itolizumab and levilimab, respectively, and 217 antibodies are in different phases of clinical trials. The authors stopped tracking this after August, 2021^1^ and no recent version of the database has been released for the community. Recently, Jaber et al. highlighted the list of ongoing clinical trials of potentially effective drugs for treating COVID-19 in the Gulf Cooperation Council (GCC) countries (9). The authors categorized the drugs in clinical trials into five categories: (a) antiviral (e.g., remdesivir, favipiravir, and darunavir); (b) antiviral repurposed (e.g., hydroxychloroquine, artemisinin, ACE Inhibitors, etc.); (c) anti-inflammatory (e.g., tocilizumab, zafirlukast, anakinra, pioglitazone, etc.); (d) anti-coagulant (e.g., bivalirudin); and (e) miscellaneous (e.g., rivaroxaban, estrogen, iloprost, etc.).

Recently, we developed a knowledgebase, COVID-19Base v2 (10), that highlights COVID-19-related biomedical entities by orchestrating natural language processing techniques, sentiment analysis, and neural networks. To the best of our knowledge, this was the first knowledgebase related to COVID-19 drugs and which also highlighted potential biomedical entities linked to COVID-19 by literature mining. To mine scientific evidence linked to COVID-19, we considered six topic-specific dictionaries (i.e., diseases, Protein Data Bank, drugs, side effects of drugs, genes, and miRNAs). In a co-occurrence-based approach, drug-disease, gene-disease, drug-PDB, and their corresponding sentences from the literature were extracted. The authors used the pre-trained model TextBlob (11), and an unsupervised model based on K-means clustering and the Word2vec model, to determine the sentiment of scores for each disease-drug pair. Subsequently, the authors identified 1,805 diseases, 2,454 drugs, and 1,910 genes associated with coronavirus-related diseases, including COVID-19, through literature mining.

With the above backdrop, it is clear that the scientific community is still trying to find an effective and well-tolerated drug for COVID-19. This ongoing global effort will require the integration of different biomedical entities that are relevant to the infection and the progression of the virus. Therefore, in this paper, we improved our knowledgebase, transitioning it from version 2 to version 3, with the overarching aim of supporting the community in their quest to find a therapeutic treatment for COVID-19. We updated our knowledgebase by adding more entities, i.e., long non-coding RNAs (lncRNAs), alternative medicines (AMs), lncRNA-disease association, and AM-disease associations. Moreover, we considered the recent corpus of literature and clinical trials to provide evidence-based support for the research community.

## 2. Materials and methods

### 2.1. Dataset collection

We examined the COVID-19 Open Research Dataset (CORD-19) (12). This dataset covers scholarly articles related to COVID-19 and other diseases related to the coronavirus family (e.g., MERS and SARS). The query “COVID-19” OR “Coronavirus” OR “Corona virus” OR “2019-nCoV” OR “SARS-CoV” OR “MERS-CoV” OR “Severe Acute Respiratory Syndrome OR Middle East Respiratory Syndrome” was used to collect literature from PubMed, PubMed Central, medRxiv, and bioRxiv. CORD-19 has accumulated literature since March 2020 and is still updating the database with relevant literature. For our study, we collected literature that was published after the vaccine rollout, starting from January 2021 up to April 2022, covering 414,899 articles with a unique “cord_uid”. We considered only the abstract of the literature corpus as we only had limited computational resources.

### 2.2. Dictionary collection

To prepare this knowledgebase, we used multiple well-known publicly available dictionaries for drug, disease, gene, miRNA, lncRNA, etc. We collected the drug names from DrugBank (13) and the side effects of drugs from SIDER (14). Disease names were collected from Disease Ontology (15). Genes, miRNAs, lncRNAs, and PDB entries were collected from HGNC (16), miRBase (17), GENCODE (18), and the Protein Data Bank, respectively. Disease-gene associations were collected from DisGeNET (19). For alternative medicines (AM), we only considered a list of entities mentioned by Jaber et al. (20) and registered with the ClinicalTrials registry^2^ up until May 2022.

### 2.3. A deep-learning model for drug-disease association

**Figure 1:**
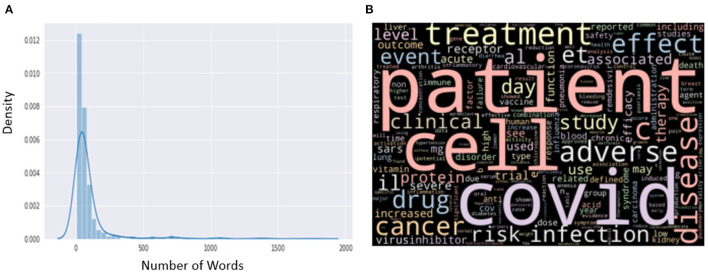
**(A)** Distribution of length from the manually annotated sentences. **(B)** Word cloud from the manually annotated sentences.

For predicting drug-disease association or AM-disease association, three independent curators reviewed 828 records from the literature and manually labeled them as positive or negative (available in GitHub). We used these records to train and validate our machine-learning model. In our training set, we manually annotated 526 sentences as positive samples (drug is reported to control the disease or there is no adverse effect of the drug on the disease) and the remaining 302 sentences as negative (the condition of the patient deteriorates or there is an adverse effect of the drug on the disease). We classified these sentences as positive or negative. The distribution plot for the length of all sentences is shown in Figure 1A, and the word cloud is shown in Figure 1B.

**Figure 2:**
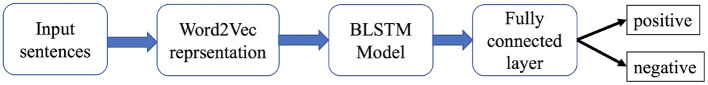
Deep-learning model architecture with a Word2Vec embedding layer with BLSTM for drug-disease association.

We used Word2vec (21) embedding to represent the sentences and bidirectional LSTM (BLSTM) (22) to build our model. For Word2vec embedding, we considered a fixed length of 400 from each sentence. The Word2Vec method takes a text corpus as input and outputs word vectors; it then creates a vocabulary from the training text input and then learns word vector representation. We made a corpus of 2,000 words. We fine-tuned the BLSTM with 15 units and a dense layer with 64 units. The architecture of our model is shown in Figure 2. The model has 640,269 parameters, of which only 39,969 were trainable parameters for BLSTM. Network parameters from the earlier layers were frozen. We divided the dataset into training and validation sets in which 20% of the dataset was used for validation purposes. We trained the model with 100 epochs with a batch size of 32. We used the Adam optimizer (23) with a learning rate of 0.01 and binary-cross entropy as a loss function. Additionally, we used early-stopping with a patience value of 40 on the validation loss.

### 2.4. Association between disease and genes and RNAs

**Figure 3:**
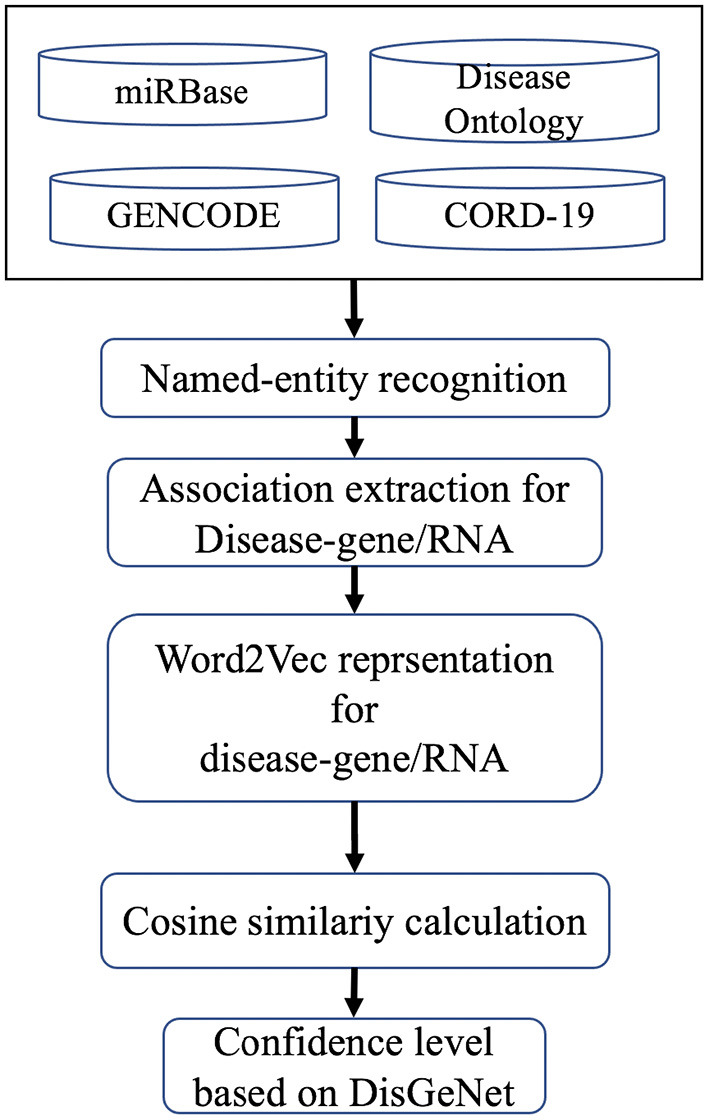
Computational workflow for finding associations between disease and genes and RNAs.

Protein coding genes, miRNAs, lncRNAs, and disease association were computed based on cosine similarity following the methodology of COVID-19Base v2 (10). Briefly, we extracted the co-occurrence of disease name and other biomedical entities (gene, miRNA, and lncRNA) in the same sentence of the corpus. Then, we represented the sentences using Word2Vec (21). We considered the associations available in DisGeNet as the gold standard and built a model to predict the association between disease and other biomedical entities (gene, miRNA, and lncRNA; Figure 3). The association score was labeled as “high,” “low,” “medium,” or “verified” based on its closet distance from the maximum, minimum, average, and verified value of DisGeNet scores, respectively. For further details see Khan et al. (10).

### 2.5. COVID-19Base web server development

**Figure 4:**
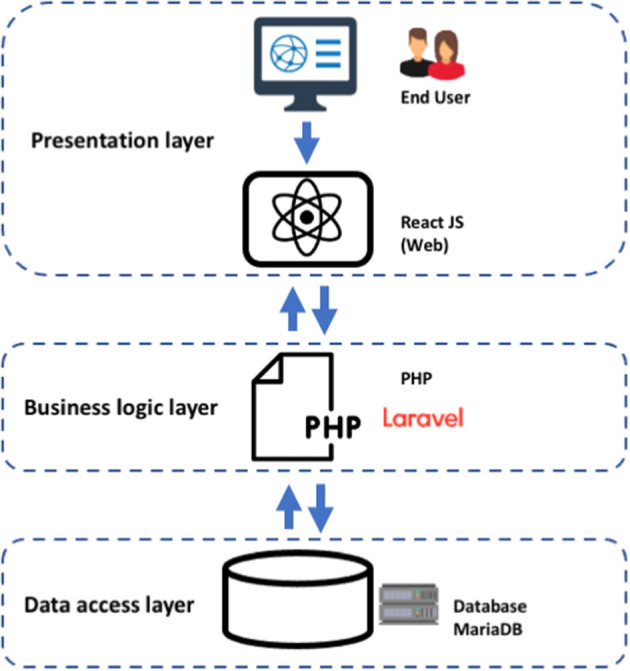
Three-tier architecture implementation for the COVID-19Base v3 webserver.

For the development of the COVID-19Base v3 web server, we considered a three-tier implementation plan, including a presentation layer, a business layer, and a persistence (database) layer. Additionally, we permitted users to be able to sort and download the query outcome from the web server. The following describes what each layer is responsible for, what technologies they involve, and how they interact with each other (Figure 4).

1. Presentation layer—user interface (UI): represents the front end of the application. It includes static content and dynamic interface elements that users see on the web page. The environment is typically a browser; the technologies used are HTML, CSS, and JavaScript. We used a React JavaScript framework to build the UI of the COVID-19Base knowledgebase. React is a declarative JavaScript structure for making dynamic client-side applications in HTML. React builds complex interfaces out of simple components, connects them to data on your backend server, and leaves them as HTML. It handles tasteful data-driven interfaces with ease. Additionally, it has all the constructs for building a modern web framework, such as great support for forms, error handling, events, and lists.
2. Business logic or application layer (BLL): part of the application backend contains the app's business logic and defines internal flows for requests and data. The environments used are a server or infrastructure as a service (IaaS), platform as a service (PaaS), or serverless cloud platforms. The programming languages used for this tier are Java, Python, PHP, JavaScript, or Ruby. We used the Laravel PHP framework to build the COVID-19Base knowledgebase.
3. Persistence or data access layer (DAL): part of the application backend that includes databases and database management systems (DBM) responsible for collecting, managing, and storing information. The environment could be servers, IaaS, PaaS, or serverless cloud platforms. The database management systems (DBMS) are MySQL, MongoDB, PostgreSQL, MariaDB, Oracle RDBMS, or Redis. We used MariaDB to build the COVID-19Base knowledgebase. MariaDB is an SQL-based database that supports ACID (atomicity, consistency, isolation, and durability)—based data processing for transactions. Atomicity ensures that every transaction is viewed as a single “unit” that totally succeeds or completely fails. If any of the statements that make up a transaction are unsuccessful, the entire transaction is unsuccessful and the database is left untouched. Consistency ensures that a transaction can only bring the database from one valid state to another valid state. Isolation ensures that the concurrent execution of transactions leaves the database in the same state as the sequential execution of the transactions. When a transaction is committed, durability ensures that it will be committed even in the event of a system failure or disaster.

## 3. Results

### 3.1. Overall statistics of the biomedical entities in COVID-19Base

Table 1 shows the overall statistics regarding the list of biomedical entities and their association pairs in our knowledgebase. In the current version (v3) of our knowledgebase, we highlighted 4,521 drugs, 2,336 diseases, 2,793 genes, 846 miRNAs, 74 lncRNAs, and 19 AMs. Additionally, we highlighted the association of 96,931 drug-disease pairs, 13,660 drug-PDB pairs, 9,346 disease-gene pairs, 4,458 disease miRNA pairs, and 290 disease-lncRNA pairs. Table 1 also highlights the associations that are related to COVID-19.

**Table 1:** Summary statistics of the knowledgebase.

**Entity**	**Count(v3)**
Drug	4,521
Disease	2,336
Gene	2,793
PDB	950
miRNA	846
lncRNA	74
AM	19
Drug-disease	96,931
Drug-PDB	13,660
Disease-gene	9,346
Disease-miRNA	4,458
Disease-lncRNA	290
COVID-19 and drug	27
COVID-19 and gene	1,315
COVID-19 and miRNA	379
COVID-19 and lncRNA	16

### 3.2. Performance of the deep-learning-based model for drug-disease associations

**Figure 5:**
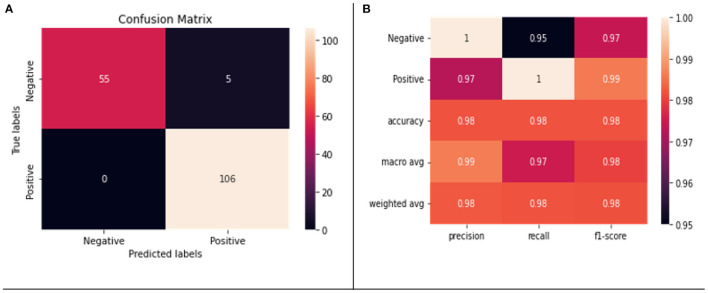
**(A)** Confusion matrix for the testing data. **(B)** Classification report on the testing data.

We applied fivefold cross validation (CV) to evaluate our model. Figures 5A, B show the confusion matrix and classification report of our model, respectively. There was no misclassification in the positive class; however, five negative samples were classified as positive samples. A weighted accuracy of 97% was achieved by the model, and >90% precision, recall, and F1-scores were achieved for both positive and negative classes.

### 3.3. Drugs extracted to the knowledgebase

In our knowledgebase, we found 4,521 unique drugs that were associated with different diseases mentioned in the CORD-19 literature. Many of these drugs were associated with COVID-19. Recently the FDA approved Paxlovid for treating COVID-19 patients. Paxlovid is reported to be 89% effective for patients with serious COVID-19 symptoms (24, 25). Ritonavir was found in our knowledgebase, which is packaged with Nirmatrelvir as Paxlovid and is effective with a positive score of 98% in the treatment of COVID-19 patients. But Paxlovid is not free from side effects (26) and extreme care should be taken prior to finalizing the proper dosage with other treatment options. Therefore, in our knowledgebase, we incorporated the list of side effects, collected from the SIDER database, for all the drugs extracted from the literature. Recently, the FDA issued an emergency use authorization for tocilizumab for treating hospitalized adult COVID-19 patients and pediatric patients of 2 years of age or more.^3^ Additionally, our computational workflow recognized tocilizumab against COVID-19, sarcoidosis, cholangitis, and different types of cancer. Thus, further investigation on this drug is warranted in the near future. Recent studies have also shown that tocilizumab reduces the risk of mechanical ventilation in hospitalized COVID-19 patients (27). Other studies showed that a tocilizumab treatment group had lower mortality compared with the control group (28), and tocilizumab-based treatment reduces the 28–30-day all-cause mortality rate, ICU admissions, superinfections, and mechanical ventilation (MV) (29). In terms of antiviral drugs in our knowledgebase, we found remdesivir and nine other antiviral drugs: favipiravir, ganciclovir, lopinavir, oseltamivir, peramivir, ribavirin, ritonavir, sofosbuvir, and umifenovir. Remdesivir was issued emergency use authorization by the FDA on the 1st May 2020 for hospitalized COVID-19 patients (30). Later, on the 22nd of October 2020, the FDA approved remdesivir for use with COVID-19 adults and pediatric patients requiring hospitalization (30). Additionally, our knowledgebase classified remdesivir as an effective drug against COVID-19, with a positive score of 95%.

### 3.4. PDB extracted to the knowledgebase

In our knowledgebase, we incorporated 950 unique PDB entries associated with 1,366 unique drugs extracted from the literature. The SARS-CoV-2 proteins that have been commonly pharmacologically targeted so far to treat COVID-19 include RNA-dependent RNA polymerase (RdRP) and main protease (Mpro). For example, Mpro has been targeted with Paxlovid^TM^, which is a combination of nirmatrelvir and ritonavir, whereas RdRP has been targeted with molnupiravir. Indeed, our analysis was able to capture these and several PDB entries found in the database belonging to these proteins. For example, one of the PDB entries, 5R7Y, represents Mpro in complex with the compound named Z45617795 and is associated with geranylgeranyl diphosphate, atazanavir, danoprevir, remdesivir, selinexor, amino acids, lisinopril, ATP, bafilomycin A1, and defibrotide in the COVID-19Base 3.0 [https://covidbase-v3.vercel.app/PDBs/5R7Y]. Similarly, the PDB entry 7BAK represents Mpro in complex with the inhibitor ebselen and is associated with ebselen, urea, trypsin, and formic acid. On the other hand, PDB entry 6WZO, which corresponds to the SARS-CoV-2 nucleocapsid dimerization domain, is associated with ivermectin, camostat, and ebselen. Additionally, we found many PDBs in our knowledgebase, such as 5REA, 6LU7, 6LZE, and 7AGA, that are related to Mpro, highlighting the continuous effort from the research community to understand the Mpro-related mechanism to inhibit the replication and infection of SARS-CoV-2 (31). Overall, our knowledgebase contains a 21,570 unique combinations of drug-PDB records, indicating the huge effort made by the research community to understand the underlying interaction mechanism between drugs and their target proteins.

### 3.5. Genes extracted to the knowledgebase

In addition to COVID-19-associated PDB entries and drug combinations, we attempted to include human genes that could be linked to other diseases mentioned in the DO database (15). Our analysis identified 2,793 human genes associated with various diseases, including those associated with cancers (such as BRCA1 and BRCA2) and cardiovascular diseases (such as PDE4D). Selecting COVID-19 under the search bar of our knowledgebase will show all genes that have been shown to be associated with this disease. So far, we have found an association of COVID-19 with 1,315 genes. Further, we have classified each such association as “low”, “medium”, and “high” to provide a better understanding of the disease-gene interaction. Additionally, we have included the abstract and the digital object identifier (DOI) of the publication that was used for the analysis. For instance, the gene ABCA3 (ATP-binding cassette subfamily A member 3, which encodes a protein that is involved in the transport of various molecules from the extracellular to the intracellular side) shows a low level of association with COVID-19 (32). The gene EPHB4 (EPH receptor B4, which encodes a protein that is involved in the development of the nervous system and other systems in humans) shows a medium level of association with COVID-19 (33). Similarly, the gene STAT1, which encodes for the STAT1 protein involved in cytokine signaling, also shows a medium level of association with COVID-19 (34).

### 3.6. MiRNAs and LncRNAs extracted to the knowledgebase

MiRNAs have been considered as molecules with a high potential for discovering drugs for COVID-19 (35). Many miRNAs associated with COVID-19 and other diseases were found in our knowledgebase. For example, the host miR-122, a liver-specific miRNA (36), has been shown to bind the SARS-CoV-2 genome (37). Additionally, miR-122 serves as a cofactor for the binding of hepatitis C virus (HCV) for its pathogenesis. Therefore, miravirsen anti-HCV RNA-based drugs should be tested against SARS-CoV-2 infection. Our knowledgebase identified miR-122 as being associated with COVID-19, suggesting the usefulness of the knowledgebase for drug repurposing. Another example is miR-21, which was shown to be effective in controlling the steatosis of HCV infection (38), as well as controlling inflammation in COVID-19 patients (39). The knowledgebase also highlighted a medium association between miR-21 and COVID-19, with a score of 0.21. Recently, cobomarsen (MRG-106), an oligonucleotide inhibitor of miR-155, was shown to be effective at controlling cellular proliferation and T-cell lymphoma (40). Additionally, our knowledgebase identified a medium association between miR-155 and COVID-19, with a score of 0.24.

Our knowledgebase summarized 16 different lncRNAs associated with COVID-19, including XIST, the lncRNA well-known for its role in X chromosome inactivation, and MALAT1 and NEAT1, two well-studied lncRNAs linked to COVID-19 (41). These lncRNAs were shown to be associated with COVID-19 with high confidence in our knowledgebase. MALAT1 has been shown to be downregulated in severe COVID-19 patients (42) and upregulated in mild COVID-19 patients (43) in CD4+ T cells. NEAT1 has been shown to be differentially expressed in bronchoalveolar lavage (BAL) cells in severe and mild COVID-19 patients (44). Differences in gender-related lethality for COVID-19 is still under investigation by many research groups (45, 46). Interestingly, the protein coding genes for the ACE2 receptor and the immune regulatory protein TLR7 are located on the X chromosome. Recently, Yu et al. suggested that the dysregulation of XIST is involved in the different immune response between men and women in COVID-19 (47).

### 3.7. Alternative medicines extracted to the knowledgebase

Alternative medicine (AM) as a treatment for disease is an area that has been relatively untapped by the scientific community. However, considering the urgency of the current pandemic and the shortage of medical facilities, AM is being considered as an adjuvant treatment plan for COVID-19 by people across the world (48). Literature mining identified 19 AMs associated with COVID-19 in our knowledgebase. As suggested by Jaber et al. (20), these AMs can be grouped into the following three categories: (a) vitamins and minerals: vitamins B, C, and D, selenium, and sodium bicarbonate; (b) dietary supplements: lactoferrin, resistant starch, and SivoMixx; and (c) herbal: cannabis/cannabidiol, Chinese preparations, fuzheng huayu, Guduchi Ghan Vati, honey, Kan Jang, Nigella/Nigella Sativa, and resveratrol. Among herbal medicines, cannabidiol, one of the constituents of cannabis, has been shown to be effective at blocking SARS-CoV-2 entry to the cell by controlling ACE2 (49). A clinical trial, NCT03944447^4^, is currently investigating the COVID-19 infection rate in medical cannabis users against the infection rate in the general population. In terms of dietary supplements, SivoMixx is being clinically trialed NCT^5^ in Italy to evaluate its efficacy in controlling acute diarrhea in COVID-19 patients. Lactoferrin is an iron-binding glycoprotein that has been shown to provide defense against pathogens (50). Multiple clinical trials^6^^,^ ^7^ are currently in progress to measure the efficacy of lactoferrin against COVID-19. Among minerals, sodium bicarbonate is historically well-known for its use against Spanish Flu in the last century (51). Two clinical trials (NCT04655716^8^ and NCT04530448^9^) are ongoing in the UK and the USA, respectively, to explore its role in the alkalinization of urine to prevent acute kidney injury resulting from progression of COVID-19. The above results indicate that there is an ongoing quest to find an adjuvant treatment plan for COVID-19 that leverages existing AMs. We believe that our knowledgebase will support the proper utilization of AMs during the pandemic based on scientific evidence.

## 4. Discussion

In the present version (v3) of COVID-19Base, we covered eight biomedical entities and their association with the scientific literature and information from ongoing clinical trials. The previous version (v2) of COVID-19Base was developed based on the literature that was published before the vaccine rollout. In COVID-19Base v2, we identified 2,454 drugs and 1,805 diseases, among other biomedical entities that were mentioned in the literature. During that period, the CORD-19 dataset was covering literature not only for COVID-19 but also for other coronavirus-related diseases, such as SARS and MERS. As a result, the numbers of entities in v2 were higher. However, after the vaccine rollout, researchers were more focused on COVID-19 disease and much of the literature in CORD-19 was focused on the discovery of therapeutic solutions. As a result, the number of unique drugs mentioned in v3 of the COVID-19Base knowledgebase increased significantly. We found 4,521 (as opposed to 2,454 in v2) drugs and 2,336 (as opposed to 1,805 in v2) diseases mentioned in the current version (v3) of COVID-19Base.

In our knowledgebase, we systematically incorporated COVID-19 and other diseases and their association with drugs and the side effects of drugs, PDB entries, protein-coding genes, miRNAs, lncRNAs, and AM. Our knowledgebase incorporates all of these biomedical entities in a single platform, making it convenient for researchers to formulate holistic ideas about all COVID-19-related entities. Moreover, we could not find any such knowledgebase in the literature regarding COVID-19.

Finally, a brief discussion on the methodical advances of COVID-19Base v3 over its predecessor (i.e., COVID-19Base v2) is in order. Among others, a notable difference lies in the use of a more sophisticated deep-learning model architecture with a Word2Vec embedding layer with a bidirectional LSTM model for drug-disease association. This is in contrast to v2, in which a relatively simple deep neural network model was used with three features, namely the minimum distance between the disease and the drug terms in the corresponding sentences, the polarity found from the pre-trained TextBlob model, and the sentiment score from an unsupervised model (based on K-means clustering and the Word2Vec model). This sophisticated model in combination with two other factors, namely a more careful manual curation for the ground truth and a more COVID-19-focused dataset (i.e., the latest version of CORD-19), resulted in a huge boost in model performance. It is important to emphasize that in COVID-19Base v3 we provided the sentiment score for each individual sentence related to drug-disease pairs. Therefore, the sentiment score for each individual sentence might look confusing considering our overall knowledge of the efficacy of a drug against COVID-19. For example, in COVID-19Base v3, many sentences related to hydroxychloroquine and COVID-19 pairs were positive as well as negative. Additionally, we know that hydroxychloroquine showed promising results at the early stage of COVID-19, but gradually over time, through multiple research experiments, it was shown to be ineffective against COVID-19 (52). Therefore, users need to consider the sentiment of each sentence in COVID-19Base v3 as a source of evidence for a particular study and should not consider it as a holistic sentiment of a drug-disease pair.

Our study has some limitations that should be addressed. We used literature from the CORD-19 dataset but there are other databases collecting COVID-19-related literature, such as LitCovid (53). Therefore, we may have missed some articles that were collected by those databases. However, overall, the coverage of the CORD-19 dataset is huge and is widely accepted in the community. Moreover, given the limitations of our computational capacity, we focused on literature abstracts, unless otherwise mentioned. However, we believe this also provides much cleaner results than the mining of full literature texts. For AM, many clinical trials have not issued a particular outcome and some of them are suspended, withdrawn, or terminated. Therefore it was difficult to reach any conclusion on the efficacy of AM from the clinical trials registry. Additionally, target structures and their interacting compounds were not linked through the PDB and PubChem databases. In future, we will consider incorporating this link to make this knowledgebase more informative.

## Data availability statement

The datasets presented in this study can be found in online repositories. The names of the repository/repositories and accession number(s) can be found in the article/supplementary material.

## Author contributions

SAB, RQ, and SM: programming and machine-learning modeling. SAB and SM: website development. TA: conception, design, funding acquisition, and original draft. All authors contributed to the writing of the manuscript and approved the submitted version.

## Footnotes

^1^ https://chineseantibody.org/opendata/dashboards/covid-19-mab-tracker

^2^ https://clinicaltrials.gov/

^3^ https://www.uptodate.com/contents/tocilizumab-drug-information?topicRef=127429&source=see_link

^4^ https://clinicaltrials.gov/ct2/show/NCT03944447

^5^ https://clinicaltrials.gov/ct2/show/NCT04368351

^6^ https://clinicaltrials.gov/ct2/show/NCT04412395

^7^ https://clinicaltrials.gov/ct2/show/NCT04427865

^8^ https://clinicaltrials.gov/ct2/show/study/NCT04655716

^9^ https://clinicaltrials.gov/ct2/show/study/NCT04530448
